# Advances in prognostic and therapeutic targets for hepatocellular carcinoma and intrahepatic cholangiocarcinoma: The hippo signaling pathway

**DOI:** 10.3389/fonc.2022.937957

**Published:** 2022-08-12

**Authors:** Geofrey Mahiki Mranda, Zhi-Ping Xiang, Jun-Jian Liu, Tian Wei, Yinlu Ding

**Affiliations:** Department of Gastrointestinal Surgery, The Second Hospital, Cheeloo College of Medicine, Shandong University, Jinan, China

**Keywords:** hippo signalling pathway, therapeutic markers, prognostic markers, hepatocellular carcinoma, cholangiocarcinoma

## Abstract

Primary liver cancer is the sixth most frequently diagnosed cancer worldwide and the third leading cause of cancer-related death. The majority of the primary liver cancer cases are hepatocellular carcinoma and intrahepatic cholangiocarcinoma. Worldwide, there is an increasing incidence of primary liver cancer cases due to multiple risk factors ranging from parasites and viruses to metabolic diseases and lifestyles. Often, patients are diagnosed at advanced stages, depriving them of surgical curability benefits. Moreover, the efficacy of the available chemotherapeutics is limited in advanced stages. Furthermore, tumor metastases and recurrence make primary liver cancer management exceptionally challenging. Thus, exploring the molecular mechanisms for the development and progression of primary liver cancer is critical in improving diagnostic, treatment, prognostication, and surveillance modalities. These mechanisms facilitate the discovery of specific targets that are critical for novel and more efficient treatments. Consequently, the Hippo signaling pathway executing a pivotal role in organogenesis, hemostasis, and regeneration of tissues, regulates liver cells proliferation, and apoptosis. Cell polarity or adhesion molecules and cellular metabolic status are some of the biological activators of the pathway. Thus, understanding the mechanisms exhibited by the Hippo pathway is critical to the development of novel targeted therapies. This study reviews the advances in identifying therapeutic targets and prognostic markers of the Hippo pathway for primary liver cancer in the past six years.

## Introduction

Worldwide, primary liver cancer is the sixth most frequently diagnosed malignancy and the third leading cause of cancer-related mortality. Primary liver malignancies comprise hepatocellular carcinoma, which accounts for 75-85% of the cases, and cholangiocarcinoma (CCA), accounting for 10-15% of the cases, plus other rare subtypes. Several risk factors for primary liver malignancies have been pointed out, ranging from metabolic diseases, viral infections, parasitic infections, food toxins, and lifestyle (1). Of note, primary liver cancer has seen a rapidly growing pace with the reported aggressive nature of the disease and difficulties in treatment (2). Most patients with primary liver cancer present with advanced disease due to the asymptomatic nature of disease. Moreover, primary liver cancers have diverse and complex molecular pathogenetic patterns that render the disease hard to treat and with high recurrences.

The Hippo pathway regulates cell proliferation and programmed cell death and maintains tissue homeostasis and stem cell function. Several upstream and downstream regulators comprising a kinase cascade chain are responsible for the functionality of the Hippo signaling system (3, 4). The core kinases include the mammalian STE20-like protein kinase (MST1/2) and the large tumor suppressor kinase 1/2 (LATS1/2). The two primary downstream regulators of the Hippo pathway are Yes-associated protein (YAP) and PDZ binding motif (TAZ). In contrast, the upstream effectors include Kidney and brain expressed protein (KIBRA), Ajuba, FAT1-4, Ras association domain family (RASSF), and Merlin. When MST1/2 binds to human Salvador 1 (SAV1), it causes phosphorylation and activation of LATS1/2. Subsequently, LATS1/2 phosphorylates and secludes YAP and TAZ by facilitating YAP/TAZ association with 14-3-3 proteins in the cytoplasm. Furthermore, MST1/2 can interact with the Monopolar spindle 1-binder kinase (MOB1), which also regulates LATS1/2 activity. YAP1 is phosphorylated and retained in the cytoplasm when the Hippo pathway is activated. Contrary, the Hippo pathway’s repression causes YAP/TAZ dephosphorylation and their translocation into the nucleus. Eventually, Yap1/TAZ interact with other transcription factors such as TEA domain (TEAD), Runt-related transcription factor (RUNX), and SMAD to induce the expression of genes such as CTGF, Survivin, CYR61, and JAG1, that facilitate migration and proliferation of tumor cells, and inhibition of apoptosis (5–12).

YAP overexpression has been associated with poor survival rates, intrahepatic metastases, vascular invasion, tumor size, diversity, and liver cirrhosis in patients with primary liver cancers (13, 14). Furthermore, among HCC experimental models, YAP/TAZ peritumoral activity tends to exhibit tumor-suppressive roles, and dual suppression of PI3KCA/YAP expression has been associated with the death of HCC and CCA cells (15, 16). Thus, this study examines publications on the Hippo signaling pathway identifying novel targets with prognostic and therapeutic potential for HCC and cholangiocarcinoma in the past six years.

## Hepatocellular adenocarcinoma

### YAP/TAZ optimization for therapeutic purposes

Reorganizing YAP/TAZ signaling and targeting Yap has been proposed to abrogate Sorafenib resistance. YAP/TAZ promotes Sorafenib-induced ferroptosis resistance *via* a TEAD-dependent route by three mechanisms, which include induced expression of SLC7A11, increased activating transcription factor 4 (ATF4) activity, and upregulation of Survivin gene expression (17, 18). Furthermore, targeting TAZ, which also regulates the BCL2L12 gene, represents a promising drug target among patients with c-myc-induced HCC patients (19). Amino acid metabolism is an essential aspect of cancer biologics. YAP and TAZ have been reported to control cancer metabolism by increasing the uptake of amino acids *via* SLC38A1 and SLC7A5 transporters, which are thought to be potential treatment targets (20). It has also been observed that, among YAP-positive malignancies, targeting α2β1 integrin and NUAK family kinase 2 (NUAK2) expression blocks tumor progression by inhibiting the MST-YAP cascade and actin-myosin activity (21–23).

Interestingly, some scientists observed that dual suppression of YAP and TAZ expression in hypoxic carcinoma cells results in increased apoptosis of cancer cells (24). The targeting of tumor lineage plasticity mechanism of HCC involving an interactive axis (CLDN6/TJP2/YAP1) has shown improved antitumor efficacy of a *de novo* anti-CLDN6 (claudin 6) monoclonal antibody conjugated to a cytotoxic agent, Mertansine DM1 (CLDN6-DM1) as a monotherapy or combined with Sorafenib (25). Lastly, the repression of TAZ expression by Diosgenin, a phytosteroid sapogenin, is reportedly an effective antitumor therapy working *via* apoptosis induction, cell migration/invasion repression, and cell proliferation inhibition (26) (**Table 1**).

**Table 1:** Summary of therapeutic and prognostic targets for HCC.

Therapeutic targets grouped by the inhibited outcomes
Apoptosis induction and inhibition of cell proliferation, migration, and cytoskeleton function BCL2L12 gene, α2β1 integrin, NUAK2, lncRNA uc.134, LOC107985656, YAP/AKT, CD44S/YAP1 feedback loop, HMGB1, MTA2, COX-2&YAP, miR-1254, miR-665, miR-186, miR-29c-3p, miR- 3127-5p, miR-590-5p, SEPT6, PLD1, MCP-1, MEIS2C, MEIS2D, YAP/NR4A1, KCTD11, FAM83D, EGFR, NATB, NEDD4/LATS1 pathway, HAUSP, CIZ1, p-Ezrin, YAP/HIF-1α, TICs, RSPON2/Hippo/YAP, S1P2, ErBB2, PI3K/AKT, JCAD/LATS1, YAP/TAZ, HIF-2α, HBXIP. Epithelial-mesenchymal-transition, vascular mimicry, cell stemness, recurrence and metastases miR-103, Frizzled 2, MORC2, USP11, Yki/YAP-Src42A/SRC, METTL3, LMO3, ACTN1, ACADL/YAP, YAP/FOXM1
Prognostic targets grouped by outcome
Poor overall, disease-free, progression-free, and relapse-free survival, early recurrence and metastases YAP/TAZ, YAP/GPX4, PDLIM1, ACTN1, ACADL, SPON2, PLG, LATS1 rs7317471, ARID1A, RDH5, MARC2, LKB1, TNFAIP8, SPRY4-AS1, TEAD, DNMT3B, Stathmin, LMNB2, ITGAV, YAP and SPH2, SOH, MAGL, Rac GTPase activating protein 1, PAI-1, YAP and FOXM1, FAM83D, NEK2,MAGL, MOB2, miR-29c-3p,USP11, KCTD11, S100A1 gene. Worst prognosis Aurora A, Aurora B Better 5-year overall survival WWC2

BCL2L12, Bcl-2-like protein 12; YAP, Yes-associated protein; HMGB1, High mobility group box 1; MTA2, Metastasis Associated 1 Family Member 2; COX-2, Cyclooxygenase-2; SEPT6, Septin 6; PLD1, Phospholipase D1; MCP-1, Monocyte Chemoattractant Protein-1; MEIS2C/D, Meis Homeobox 2C/D; NR4A1, Nuclear receptor subfamily 4 group A member 1; KCTD11, Potassium Channel Tetramerization Domain Containing 11; FAM83D, Family with Sequence Similarity 83 Member D; NATB, NatB-mediated protein N-α-terminal acetylation; NEDD4, Neuronally Expressed Developmentally Downregulated 4; LATS1, Large tumor suppressor kinase 1; HAUSP, Human deubiquitinating Enzyme; CIZ1, Cip1-interacting zinc finger protein; p-Ezrin, Phosphorylated Ezrin; TICS, Tumor-initiating cells; RSPON2, R-spondin-2 precursor; S1P2, Sphingosine 1-phosphate receptor 2; ErBB2, Erb-B2 receptor Tyrosine Kinase 2; PI3K, Phosphoinositide 3-kinase; JCAD, Junctional Cadherin 5 Associated with coronary artery disease; HIF, Hypoxia-inducible factor; HBXIP, Hepatitis B X-interacting protein; MORC2, MORC Family cw-Type Zinc Finger 2; USP11, Ubiquitin-specific protease 11; METTL3, Methyltransferase 3, N6-Adenosine Methyltransferase Complex Catalytic Subunit; ACADL, Acyl-CoA Dehydrogenase Long Chain; GPX, Glutathione Peroxidase 1; PDLIM1, PDZ and LIM Domain 1; SPON2, Spondin 2; PLG, Plasminogen; ARID1A, AT-Rich interaction Domain 1A; RDH5, Retinal dehydrogenase 5; MAGL, Monoacylglycerol Lipase; NEK2, Never in mitosis gene-A-related kinase 2; TEAD, TEA Domain Transcription factor 1; TNFAIP8, TNF Alpha Induced Protein 8; MARC2, Mitochondrial Amidoxime Reducing Component 2; LKB1, Liver kinase B1; DNMT3B, DNA methyltransferase 3 beta; LMNB2, Lamin B2; ITGAV, Integrin Subunit Alpha V; SPH2, S-protein homolog 2; PAI-1, Plasminogen activator inhibitor 1; WWC2, WW and C2 Domain Containing 2.

### Significant long chain noncodingRNAs (lncRNAs) in HCC treatment

Long-chain noncoding RNAs play crucial roles in regulating microRNAs that are involved in carcinogenesis. The distortion of their activity as regulators of cancer predisposes to cancer growth. Thus, novel lncRNAs mediating their effects by CUL4A-mediated ubiquitination of large tumor suppressor kinase 1 (LATS1), enhancing YAP^S127^ phosphorylation, and activating the tumor-suppressive Hippo pathway (miR-106b-5p/LATS1) have been deemed potential HCC treatment options leading to tumor growth inhibition (27, 28) (**Table 1**).

### Drugs and plant derivatives with therapeutic actions on HCC

**Figure 1:**
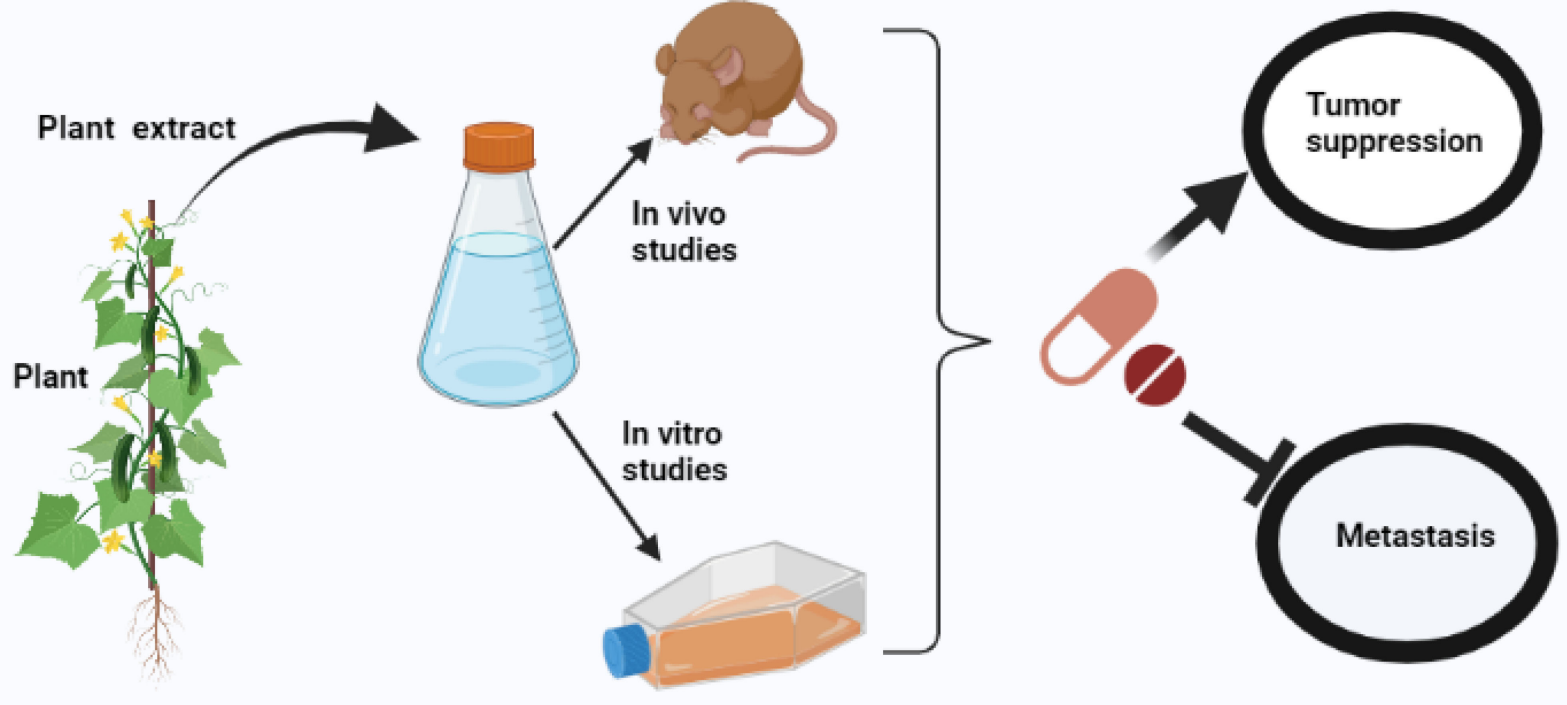
Illustrating the activity of antitumor drugs in a mouse model and human primary liver cancer cells.

Drugs such as Metformin, Artemisinin, Evodiamine, tankyrase inhibitors, Xiaoping, Wogonin, statin, and Dercusin have been deemed promising agents in treating HCC. These drugs exert their antitumor effects *via* mechanisms such as inhibition of IL-2 and LATS1 expression, Mst1/2 activation, and upregulation of LATS1 phosphorylation, N-cadherin-Snail-E-cadherin axis regulation, proliferation suppression, apoptosis induction, angiomotin-like protein 1/2 (AMOTL1/2) upregulation, Hippo, Wnt, hedgehog pathway, and cell cycle inhibition (**Figure 1**). Statins consumption, in particular, is associated with prolonged recurrence-free survival (29–36). In research reports from other investigators, several drug combinations with improved antitumor efficacy exhibited tumor growth suppression and enhanced apoptosis, such as a pan-inhibitor of Aurora Kinases (SNS-314) and Hippo pathway inhibitors and Hypocrellin A and Oleanolic acid, have been proposed (37, 38). Furthermore, in myc/Ras-induced HCC, a combination of tadalafil (a phosphodiesterase 5 inhibitor) and JQ1 (Bromodomain and Extra-Terminal domain inhibitor) evades the BET inhibitor’s resistance influenced by YAP/TAZ expression (39). The antitumor activity of Cisplatin in HCC improved when it was administered with Melatonin due to downregulation of YAP and caspase-3 and poly ADO-ribosome polymerase cleavage (40).

Moreover, targeting the S100A1 gene seemed to enhance Cisplatin’s inhibitory effects (41). Reportedly, reactive oxidative species (H2O2)-induced actuation of YAP1 by the c-Myc pathway represents a possible treatment option that rejuvenates the unfolded protein pathway (42) (**Table 2**). Recently, α-hederin has been reported as an agonist of the Hippo pathway that augments effects such as apoptosis, proliferation inhibition, YAP nuclear levels reduction, and upregulates the Hippo pathway-related proteins and genes. Notably, α-hederin suppressed tumor growth and weight in the mouse model (43). Finally, targeting RNA-binding protein Dnd1 suppresses spheroid formation and expression of stemness-related genes and enhances Sorafenib sensitivity making it a probable drug target for HCC treatment (44) (**Table 2**).

**Table 2:** Summary of therapeutic and prognostic targets for Cholangiocarcinoma.

Therapeutic targets grouped by inhibited outcomes
Apoptosis induction and inhibition of cell proliferation, migration, and cytoskeleton function Mcl-1, FGFR, PDGFR, MNX1-AS1, FOXM1, G9a, MFAP5, TAZ, HPR lcnRNA, YAP Metastasis miR-29-3p, Piezo 1 mechanosensitive ion channel, Agrin
Prognostic targets
Poor overall and disease-free survival, early recurrence, metastases LCK, circACTN4, YAP/TAZ, Agrin, DEPDC1, FUT4, MDK, PACS1, PIWIL4 genes, miR-22, miR-551b, cg27362525 and cg26597242 CpG

Mcl-1, Myeloid cell leukemia factor 1; FGFR, Fibroblast Growth Factor Receptor; PDGFR, Platelet-derived growth factor receptor alpha; FOXM1, Forkhead box M1; G9a, histone methyltransferase G9a; HPR, Hippo-pathway-related; LCK, Lymphocyte-specific protein tyrosine kinase; circACTN4, Circular Alpha-actinin-4-Homo sapiens; DEPDC1, DEP Domain Containing 1; FUT4, Fucosyltransferase 4; MDK, Midkine; PACS1, Phosphofurin acidic cluster sorting protein 1; PIWIL4, Piwi-like protein 4-Homo sapiens.

### Significant interacting pathways for HCC treatment

Interacting pathways play critical roles in hepatocellular carcinogenesis, and their optimization provides opportunities for developing targeted therapeutic strategies. The inhibition of YAP/AKT in the Hippo/PI3K-PTEN-mTOR pathways and regulation of the Hippo/YAP and PI3K/AKT pathways are associated with induced apoptosis and suppressed tumor growth by FR5 compound and poplar propolis extract (45, 46). Blockage of a feedback loop involving CD44S and YAP1 (CD44S regulates YAP expression *via* PI3K/AKT pathway and YAP/TEAD axis regulates CD44S) inhibits vascular invasion and more severe form of liver cirrhosis (47). Furthermore, utilizing genetic or pharmacologic blockage involving HMGP1/YAP/HIGF1α, MTA2-FRDM6-Hippo, COX-2-PGE_2_-EP2-Gαs-β-catenin-YAP-COX-2, and their respective targets (i.e., high mobility group box one protein (HMGB1), metastatic associated protein 2 (MTA2), cyclooxygenase 2 (COX-2) &YAP) prevents tumorigenesis, excessive glycolysis, and metastases. Lastly, utilizing a FUS-LATS1/2 axis inhibited HCC progression by activating the Hippo pathway (48–51) (**Table 1**).

### MicroRNAs in HCC treatment

The upregulation of some microRNAs is associated with tumor proliferation, epithelial-mesenchymal transition, and metastases. Some recently documented mechanisms for these events include inactivation of the Hippo/Yap *via* paired box 5 (PAX5), Protein tyrosine phosphatase receptor type B (PTPRB) downregulation, and LATS2 inhibition. Thus, targeting specific miRNA molecules (i.e. miR-1254, miR-665, miR-103) propagating tumorigenesis provides novel drug options against HCC (52–54). Nonetheless, the overexpression of other downregulated miRNAs is associated with inhibiting hepatocarcinogenesis *via* downregulation of YAP1, DNA methyltransferase 3 beta (DNMT3B) upregulation leading to LATS1 methylation, S-phase arrest through upregulating p21 and p27 expression, and inhibiting PI3K/AKT pathway (55–57). Reportedly, a microRNA-590-5p represses Adriamycin chemoresistance *via* Yap expression regulation (58). Noteworthy, miR-21 deficiency is associated with tumorigenesis through increased oncogenes expression and minute dysregulation of the Hippo signaling pathway, signal transducer and activator of transcription factor 3 (STAT3), and mitogen-activated protein kinase (MAPK) pathways. Hence, prudence is recommended in adopting miR-21 inhibitors in treating liver cancer (59) (**Table 1**).

### Proposed therapeutic targets for HCC

Seemingly, Septin 6 (SEPT6), Frizzled-2, MORC Family cw-Type Zinc Finger 2 (MORC2), Ubiquitin-specific protease 11 (USP11), Yki/Yap-Src42A/SRC positive feedback loop, m6A methyltransferase 3 (METTL3), and family with sequence similarity 83 member D (FAM83D) play crucial roles in HCC tumorigenesis by promoting vascular mimicry, cell stemness, migration, invasion, and silencing of the Hippo pathway by DNA methylation-dependent mechanism. Targeting these genes shows prospective benefits such as preventing disease progression, recurrence post-transplantation, and metastases. Besides, USP11 overexpression is linked with a 5-fold risk of all-cause-related mortality (60–65). Furthermore, other crucial targets and their regulatory loops representing potential therapeutic options for HCC have been reported, including Phospholipase 1 inhibited by T-box transcription factor 3 (TBX3), YAP-dependent monocyte chemoattractant protein 1 (MCP-1) in a protumoral microenvironment, Meis homeobox 2C/D (MEIS2C/D) activating Wnt/β-catenin and inhibiting Hippo pathway, and the YAP/nuclear receptor 4A1 (NR4A1) (66–69) (**Table 1**).

Strategically, the combined use of inhibitors of YAP and epidermal growth factor receptor (EGFR) targeting the EGFR-PI3K-PDK1 pathway shows improved cytotoxicity for HCC cells (70). Optimizing the tumor-suppressive effects of potassium channel tetramerization domain containing 11 (KCTD11) (by p21 activation and suppression of cell cycle proteins) and LATS1 overexpression with YAP1 nucleocytoplasmic translocation by tumor growth factor-beta 1 (TGF- β1) inhibit HCC cells growth and development (71, 72). Further, the suppression of LIM domain only 3 (LMO3) expression exerting its actions *via* the LATS1/Hippo pathway evades invasion and metastasis by cancer cells (73). Otherwise, actinin alpha 1 (ACTN1) expression, acyl-CoA dehydrogenase Long-chain (ACADL)/YAP, and YAP/Forkhead Box M1 (FOXM1) are proposed targets for preventing tumor growth and early recurrence of HCC (74–76) (**Table 1**).

The inhibition of cell growth, migration, and proliferation, as well as disruption of cytoskeleton function, prevent tumorigenesis. Accordingly, several investigators have revealed that targeting NatB-mediated protein N-α-terminal acetylation (NATB) expression, Neuronally Expressed Developmentally Downregulated 4 (NEDD4)/LATS1 pathway, Herpesvirus-associated ubiquitin-specific protease (HAUSP) expression, Cip1-interacting zinc finger protein 1 (CIZ1) expression, and phosphorylated-ezrin (p-Ezrin) effectively arrested carcinogenesis in HCC (77–81). Further, YAP targeting has shown suppression of cancer cell growth in patients with hypoxia-mediated HCC metabolism and HBX-induced HCC (82, 83). Specific targeting of tumor-initiating cells (TICs) seems to overcome resistance to antiangiogenic therapy in HCC. Moreover, TICs have been shown to recruit tumor-infiltrated type II macrophages in the early phase; thus, suppressing TICs *via* YAP or M2 macrophages is a valuable treatment option in HCC (84, 85) (**Table 1**).

Several other targets inhibit HCC cells proliferation, growth, and migration and induce apoptosis through different mechanisms such as RSPO2/Hippo/Yap and S1P2-induced Yap activation, EGF-induced Erb-B2 receptor Tyrosine Kinase 2 (ErBB2) and PI3K/AKT activation, and Junctional Cadherin 5 Associated with coronary artery disease (JCAD)/LATS1 interaction (86–89). Furthermore, targeting Hepatitis B X-interacting protein (HBXIP), which potentiates its effects by upregulating YAP through the transcription factor c-myb coactivation in HCC cells, prevents cancer cell proliferation (90). Succinctly, Hypoxia-inducible factor-2α is a potential antitumor target that facilitates NASH-induced hepatocarcinogenesis progression, and HIF-2α inhibitors reportedly block this activity (91). Recently, suppression of STK25 expression in HCC cell lines has been proposed as a new treatment target among cancers expressing miR-4800-3p (92). Lastly, exploring potential Scrib agonists may provide potent antitumor drugs as Scrib expression inhibits tumor cell proliferation *via* repression of Yap, c-Myc, and cyclin D1 (93) (**Table 1**).

### Prognostic markers for progression, survival, recurrence or metastasis

Cytoplasmic YAP and nuclear TAZ expression in Keratin 19 negative HCC patients is associated with poor overall and disease-free survival (94). The overexpression of YAP leads to Plasminogen activating inhibitor-1 (PAI-1) overexpression, which is associated with poor survival and early recurrence rates (23). High YAP and low glutathione peroxidase 4 (GPX4) expression are associated with Sorafenib treatment’s increased survival. Further, YAP signaling modifications present a potential biomarker for tumor ferroptosis-induced response prediction (95). Nevertheless, YAP and Src homology phosphotyrosine phosphatase 2 (SHP2) expression represents unfavorable prognostic indicators with poor overall and recurrence-free survival (96). Other scientists also observed that overexpression of c-Src had a negative correlation with patient survival (97). A group of investigators noted that Yap/TAZ expression in HCC was associated with high serum alpha-fetoprotein levels, increased proliferation activity, microvascular invasion, and stemness and epithelial-mesenchymal transition-related expression markers such as SMAD2/3, CAIX, and p53. Consequently, they proposed consideration for the status of the hypoxia markers when using YAP/TAZ to determine the behavior of HCC (98). Notably, a prognostic nomogram based on five Hippo-related genes (i.e., the master regulator of cell cycle and proliferative metabolism (MYC), neurofibromatosis 2 (NF2), misshapen-like kinase 1 (MINK1), baculoviral IAP repeat-containing 3 (BIRC3), and casein kinase 1 epsilon (CSNK1E)) has been proposed, outperforming available clinical parameters in the prognostication of HCC (99). PDZ and LIM domain 1 (PDLIM1), ACTN1, mitochondrial ACADL, and matricellular spondin-2 (SPON2) expressions involved in mechanisms such as enhancing the Hippo pathway activity, reducing RhoA GTPases activity, repressing cell proliferation, repressing tumor growth, facilitating interaction with MOB1, decreasing phosphorylation of LATS1 and YAP, M1-macrophage recruitment facilitation, and suppression hepatocellular carcinoma metastases have been implicated in prognosticating HCC (75, 100–102) (**Table 1**).

Among HBV-induced HCC patients, Plasminogen expression inhibits cell apoptosis and enhances cell line growth through upregulation of the SRC gene and the inhibition of the Hippo signaling pathway (103). Expression of a genetic variant of LATS1 (LATS1 rs7317471) in HCC patients exhibiting age below 53 years, female gender, smoking, alcohol drinking, and Barcelona clinic liver cancer stage B is associated with decreased risk of death (104). Low AT-Rich interaction Domain 1A (ARID1A), downregulation of Retinal dehydrogenase 5 (visual cycle enzyme), and repressed Mitochondrial Amidoxime Reducing Component 2 (MARC2) expression have been associated with poor overall and disease-free survival, metastasis, and disease progression. These observations were mediated *via* mechanisms, including; immune activity regulation, regulation of genes related to HCC development, regulation of the epithelia-mesenchymal-transition process, regulation of p27 levels, and regulation of HNF4A expression (105–107). Loss of Liver kinase B1 expression correlates with migration and invasion of liver cancer cells *via* the ZEB1-induced Yap signaling (108).

Meanwhile, overexpression of genes related to immune infiltration, actin stress fiber congregation, cell migration, and invasion leads to poor overall clinical outcomes and disease-free survival (109, 110). TNF Alpha Induced Protein 8 (TNFAIP8), novel enhancer RNA (SPRY4-AS1), TEA Domain Transcription factor 1 (TEAD), DNMT3B, and Stathmin overexpression were associated with recurrence, poor disease-free, progression-free, relapse-free, and overall survival. These genes propagated the poor outcomes *via* mechanisms such as inhibition of YAP phosphorylation, decreasing LATS1 phosphorylation, increasing iron accumulation and consequent oxidative injury, and activity of several infiltrating immune cells (111–115). Researchers noted that FAM83D and NEK2 genes were related to high recurrences, poor survival, and metastases following liver transplantation and hepatectomy. Apart from MAPK and TGF-beta, FAM83D enhanced CD44 expression and CD44-cancer stem cell malignancy. Regarding NEK2, mechanistic studies revealed that EMT was essential in NEK2-induced HCC cell invasion (116, 117). Furthermore, an interplay between YAP and FOXM1 leading to chromosomal instability has been associated with poor survival, and early recurrences. Otherwise, inactivation of the Hippo pathway has been linked to overall poor prognosis (76, 118) (**Table 1**).

The overexpression of Rac GTPase activating protein 1 is reportedly associated with shorter survival from enhanced cytokinesis and suppressed apoptosis. Aurora A/B co-overexpression is associated with the worst prognosis among HCC patients (37, 119). Monoacylglycerol lipase overexpression has been linked with proliferation and invasion of HCC cells *via* Prostaglandin E2 and Lysophosphatidic acid mechanisms (120). Mps one binder kinase activator-like 2 (MOB2) expression influences migration and invasion of cancer cells and may represent a valuable marker of disease progression (121). Notably, silencing of Hippo signature (SOH) is associated with a poorer prognosis than non-silencing in HCC patients. SOH was determined as an independent predictor of poor prognosis on multivariate analysis (118). Nonetheless, the expression of WW and C2 Domain Containing 2 (WWC2) is associated with better 5-year overall survival among HCC patients (122) (**Table 1**).

## Cholangiocarcinoma

### Proposed therapeutic targets for CCA treatment

Identifying new molecular targets that may potentially improve the treatment of cholangiocarcinoma has been an unceasing expedition (123). Furthermore, molecules that propagate cancer cell growth, migration, metastasis, or proliferation are indispensable in achieving intent to cure goals. As such, investigators observed that depleting myeloid leukemia 1 (Mcl-1) expression is associated with increased cell death in CCA cells. Accordingly, administering a pan-Fibroblast growth factor receptor (FGFR) inhibitor in YAP expressing cells was associated with cancer cell death. Similar effects were observed with the inhibition of the platelet-derived growth factor receptor (PDGFR) in YAP-expressing cells. Thus, YAP expression may be adopted in assessing FGFR therapies response, and the PDGFR-SFK cascade regulating YAP activation presents a novel treatment strategy (124, 125). Targeting LncRNA MNX1-AS1 expression, which exerts its actions *via* MNX1-AS1/c-Myc and MAZ/MNX1/Ajuba/Hippo pathway, correlates with tumor growth, and migration, and metastasis inhibition (126). Moreover, inhibiting genes that downregulate the miR-29-3p family or upregulation of transcription factor SP1 may prevent the malignant transformation of ICC cells by expressing ITGA6/ITGB1 genes (127) (**Table 2**).

The inactivation of a mechanosensitive ion channel Piezo 1 (acting through the Hippo/YAP axis) and its downstream effectors, and the inhibition of expression of several other genes, including; FOXM1 (regulator of CIN25 gene), Agrin, histone methyltransferase G9a, Microfibril associated protein 5 (MFAP5) (transcriptional target of YAP/TEAD), and TAZ appears to prevent metastases, induce cell death, and suppress proliferation. Moreover, targeting these molecules inhibited colony formation, migration, invasion, tumor angiogenesis, and enhanced vitamin D3-sensitivity *via* the p53/CYP24A1 pathway. Some of the mechanisms identified include decreased H3K9me2, restoration of LATS1, YAP activity inhibition, and TAZ inhibition (128–133). Furthermore, inhibition of a Hippo-pathway-related long noncoding RNA that interacts with mTORC1 subunit Raptor is associated with suppression of tumor growth, and YAP/TAZ-directed therapies have shown benefits in treating CCA patients with chromosomal instability (134, 135) (**Table 2**).

### Drugs and plant derivatives for tumor growth suppression and metastasis prevention

Licochalcone A compound and antiparasitic macrolide lactones (AML) combined with TGF- β pathway inhibitor repress Yap expression and transcriptional tendency *via* separate mechanisms that ultimately prevent tumor growth. Licochalcone A suppresses PES1 expression and nuclear localization while AML targets YAP/TAZ activity (136, 137). A group of investigators reported improved chemosensitivity of conventional therapies for CCA when administered concomitantly with histone deacetylase (HDAC) inhibitor that also allowed dose reduction. The drugs used in the study were gemcitabine, cisplatin, 5-fluorouracil (5-FU), oxaliplatin, or gemcitabine plus cisplatin (138),. Further, decreased phosphatase SPH2 activity in cholangiocarcinoma patients can induce chemotherapy resistance through the MCL1-mediated pathway. Thus, targeting the MCL1 pathway provides promising treatment alternatives for patients exhibiting chemotherapy resistance from low SPH2 (139) (**Table 3**).

**Table 3:** Summary of drugs and plant derivatives for HCC and Cholangiocarcinoma treatment.

Hepatocellular carcinoma
Metformin→ Induces apoptosis, inhibits proliferation, migration and invasion *via* IL-2 inhibition and LATS1/2 inhibition (29) Evodiamine→ Inhibits proliferation and induces apoptosis *via* MST1/2 activation and upregulation of LATS1 phosphorylation (31) Artemisinin→ Suppresses cancer cells growth, migration and invasion *via* N-cadherin-Snail-E-cadherin axis regulation (30) Tankyrase Inhibitors→ downregulates YAP/TAZ *via* AMOTL1/2 upregulation (32) Xiaoping→ Inhibits Hippo, Wnt and Hedgehog pathways and decreased stemness markers and totipotency factors expression (33) Wogonin→ Induces cell cycle arrest and apoptosis *via* MOB1/LATS1 signaling activation (34) Statin→ Induces apoptosis and proliferation suppression *via* TAZ suppression (35) Decursin→ Induces apoptosis *via* LATS1/βTRCP degradation of YAP1 (36) Hypocrellin A and Oleanolic acid→ Suppresses tumor growth *via* Hippo/YAP (38) Apoptosis induction *via* Aurora Kinases/YAP/P21 axis suppression (37) Proposed combination of Aurora kinases inhibitors and Hippo pathway inhibitors Targeting tumor lineage plasticity mechanism (CLDN6/TJP2/YAP1 interacting axis) (25) A *de novo* anti-CLDN6 monoclonal antibody conjugated to a cytotoxic agent →Mertansine DM1 Overcoming chemotherapeutic resistances YAP/TAZ-induced BET inhibitors resistance in Myc/Ras-induced HCC (39) Combine tadalafil(PDE5 inhibitor)+ BET inhibitor Cisplatin-resistance Melatonin-via YAP downregulation (40) S100A1 gene targeting (41) Sorafenib-resistance Target YAP/TAZ and ATF4→inhibit ferroptosis resistance and Survivin expression (17) Target RNA-binding protein Dnd1 (44) Antiangiogenic-resistance Target Tumor-initiating cells (84) Adriamycin-resistance Target microRNA-590-5p expression (58)
Cholangiocarcinoma
Licochalcone A → Inhibit of cell growth through Hippo pathway *via* PES1 suppression (136) Inhibition of proliferation and cellular migration *via* YAP/TAZ repression (137) Antiparasitic macrolide lactone + TGF-β pathway inhibitors Overcoming Gemcitabine-resistance (138) HDAC inhibitor-induce apoptosis through targeting Hippo pathway *via* miR-509-3p expression

AMOTL, Angiomotin Like; BET, Bromodomain and extra-terminal domain; ATF4, Activating transcription factor 4; PES1, Pescadillo ribosomal biogenesis factor 1; TGF- β, Transforming growth factor beta; HDAC, Histone deacetylase; MOB1, MOB Kinase Activator 1A; βTRCP, beta-Transducin Repeat Containing E3 Ubiquitin Protein Lipase; MST1/2, Mammalian-sterile like 1/2; PDE5, Phosphodiesterase 5.

### Prognostic markers for survival, recurrence, and metastases

Among post-radical CCA resection patients, high Lymphocyte-specific protein tyrosine kinase (LCK) and Circular Alpha-actinin-4 (circACTN4) expression are related to early tumor recurrence and worse prognosis, respectively. High circACTN4 expression is associated with a worse prognosis as it enhances proliferation and metastases by molecular miR-424-5p sponging and interacts with Y-box homolog protein 1 (74, 140). Furthermore, YAP/TAZ dual positivity following tumor resection correlates with poor overall and disease-free survival typified by worse TNM stages, poor tissue differentiation, and high CA19-9 levels (141). Besides, YAP overexpression in CCA is associated with poor overall survival (142, 143). Lastly, DEP Domain Containing 1 (DEPDC), Fucosyltransferase 4 FUT4, Midkine (MDK), Phosphofurin acidic cluster sorting protein 1 (PACS1), Piwi-like protein 4-Homo sapiens 1 (PIWIL4) genes, miR-22, miR-551b, and cg27362525 and cg26597242 CpG have been proposed as potential prognostic markers for cholangiocarcinoma (144) (**Table 2**).

## Conclusion

Although primary liver cancer remains challenging in its management, the newly proposed targets potentiating their effects *via* tumor cell viability, proliferation, migration, and apoptosis holds promising outcomes in treating and prognosticating the disease. Furthermore, targeting the identified molecules in this study led to inhibition of tumor cell growth and migration and activated the apoptosis of the tumor cells. Additionally, the new targets effectively predicted the prognosis of patients with primary liver cancer in terms of metastases risk, disease-free, progression-free, and overall survival. Currently, YAP and TAZ expression serve that purpose. The discovery of new targets should be coupled with developing novel nomograms that are key to predicting the prognosis in primary liver cancer patients. These nomograms should incorporate individual risk factors likely to influence treatment outcomes in different patients. Furthermore, it is commendable to validate further the significance and applicability of these new targets identified as a critical phase towards their drugability trials.

## Author contributions

GM designed and drafted the manuscript. GM, Z-PX, J-JL, TW, and YD discussed and revised the manuscript. All authors contributed to the article and approved the submitted version.

## Acknowledgments

The authors extend their gratitude to the working staff of the Gastrointestinal Surgery Department of The Second Hospital of Shandong University.

## Conflict of interest

The authors declare that the research was conducted in the absence of any commercial or financial relationships that could be construed as a potential conflict of interest.

## Publisher’s note

All claims expressed in this article are solely those of the authors and do not necessarily represent those of their affiliated organizations, or those of the publisher, the editors and the reviewers. Any product that may be evaluated in this article, or claim that may be made by its manufacturer, is not guaranteed or endorsed by the publisher.

## Glossary

**Table:**

BCL2L12	Bcl-2-like protein 12
YAP	Yes-associated protein
HMGB1	High mobility group box 1
MTA2	Metastasis Associated 1 Family Member 2
COX-2	Cyclooxygenase-2
SEPT6	Septin 6
PLD1	Phospholipase D1
MCP-1	Monocyte Chemoattractant Protein-1
MEIS2C/D	Meis Homeobox 2C/D
NR4A1	Nuclear receptor subfamily 4 group A member 1
KCTD11	Potassium Channel Tetramerization Domain Containing 11
FAM83D	Family with Sequence Similarity 83 Member D
NATB	NatB-mediated protein N-a-terminal acetylation
NEDD4	Neuronally Expressed Developmentally Downregulated 4
LATS1	Large tumor suppressor kinase 1
HAUSP	Human deubiquitinating Enzyme
CIZ1	Cip1-interacting zinc finger protein;
p-Ezrin	Phosphorylated Ezrin
TICS	Tumor-initiating cells
RSPON2	R-spondin-2 precursor
S1P2	Sphingosine 1-phosphate receptor 2
ErBB2	Erb-B2 receptor Tyrosine Kinase 2
PI3K	Phosphoinositide 3-kinase
JCAD	Junctional Cadherin 5 Associated with coronary artery disease
HIF	Hypoxia-inducible factor
HBXIP	Hepatitis B X-interacting protein
MORC2	MORC Family cw-Type Zinc Finger 2
USP11	Ubiquitin-specific protease 11
METTL3	Methyltransferase 3, N6-Adenosine Methyltransferase Complex Catalytic Subunit
ACADL	Acyl-CoA Dehydrogenase Long Chain
GPX	Glutathione Peroxidase 1
PDLIM1	PDZ and LIM Domain 1
SPON2	Spondin 2
PLG	Plasminogen
ARID1A	AT-Rich interaction Domain 1A;
RDH5	Retinal dehydrogenase 5
MAGL	Monoacylglycerol Lipase
NEK2	Never in mitosis gene-A-related kinase 2
TEAD	TEA Domain Transcription factor 1
TNFAIP8	TNF Alpha Induced Protein 8
MARC2	Mitochondrial Amidoxime Reducing Component 2
LKB1	Liver kinase B1
DNMT3B	DNA methyltransferase 3 beta
LMNB2	Lamin B2
ITGAV	Integrin Subunit Alpha V
SPH2	S-protein homolog 2
PAI-1	Plasminogen activator inhibitor 1
WWC2	WW and C2 Domain Containing 2
Mcl-1	Myeloid cell leukemia factor 1
FGFR	Fibroblast Growth Factor Receptor
PDGFR	Platelet-derived growth factor receptor alpha
FOXM1	Forkhead box M1
G9a	histone methyltransferase G9a
HPR	Hippo-pathway-related
LCK	Lymphocyte-specific protein tyrosine kinase
circACTN4	Circular Alpha-actinin-4-Homo sapiens
DEPDC1	DEP Domain Containing 1
FUT4	Fucosyltransferase 4
MDK	Midkine
PACS1	Phosphofurin acidic cluster sorting protein 1
PIWIL4	Piwi-like protein 4-Homo sapiens
AMOTL	Angiomotin Like
BET	Bromodomain and extra-terminal domain
ATF4	Activating transcription factor 4
PES1	Pescadillo ribosomal biogenesis factor 1
TGF- b	Transforming growth factor beta
HDAC	Histone deacetylase
MOB1	MOB Kinase Activator 1A
bTRCP	beta-Transducin Repeat Containing E3 Ubiquitin Protein Lipase
MST1/2	Mammalian-sterile like ½
PDE5	Phosphodiesterase 5.
MYC	Master regulator of cell cycle and proliferative metabolism
NF2	Neurofibromatosis 2
MINK1	Misshapen-like kinase 1
BIRC3	Baculoviral IAP repeat containing 3
CSNK1E	Casein kinase 1 epsilon
